# Algorithms and Results of Eye Tissues Differentiation Based on RF Ultrasound

**DOI:** 10.1100/2012/870869

**Published:** 2012-05-02

**Authors:** R. Jurkonis, A. Janušauskas, V. Marozas, D. Jegelevičius, S. Daukantas, M. Patašius, A. Paunksnis, A. Lukoševičius

**Affiliations:** ^1^Biomedical Engineering Institute, Kaunas University of Technology, Studentu Street 65, 51369 Kaunas, Lithuania; ^2^JSC “Stratelus”, Naugarduko Street 3, 03231 Vilnius, Lithuania; ^3^Department of Ophthalmology, Institute of Neurosciences, Academy of Medicine, Lithuanian University of Health Sciences, Eiveniu Street 4, 50009 Kaunas, Lithuania

## Abstract

Algorithms and software were developed for analysis of B-scan ultrasonic signals acquired from commercial diagnostic ultrasound system. The algorithms process raw ultrasonic signals in backscattered spectrum domain, which is obtained using two time-frequency methods: short-time Fourier and Hilbert-Huang transformations. The signals from selected regions of eye tissues are characterized by parameters: B-scan envelope amplitude, approximated spectral slope, approximated spectral intercept, mean instantaneous frequency, mean instantaneous bandwidth, and parameters of Nakagami distribution characterizing Hilbert-Huang transformation output. The backscattered ultrasound signal parameters characterizing intraocular and orbit tissues were processed by decision tree data mining algorithm. The pilot trial proved that applied methods are able to correctly classify signals from *corpus vitreum* blood, extraocular muscle, and orbit tissues. In 26 cases of ocular tissues classification, one error occurred, when tissues were classified into classes of *corpus vitreum* blood, extraocular muscle, and orbit tissue. In this pilot classification parameters of spectral intercept and Nakagami parameter for instantaneous frequencies distribution of the 1st intrinsic mode function were found specific for *corpus vitreum* blood, orbit and extraocular muscle tissues. We conclude that ultrasound data should be further collected in clinical database to establish background for decision support system for ocular tissue noninvasive differentiation.

## 1. Introduction

Ultrasound scanning of eye is a well-known instrumental investigation [1–3]. Ultrasound B-scans help to visualize internal structure of the tissues. In ophthalmology, B-scans are used to show cross-sectional view of the eye by displaying an image of ultrasound signal intensities originating from nonhomogeneities within tissue. The ultrasound methods combined with optical methods are of high importance in diagnosis and management of eye tumors [4, 5]. Limited set of B-scan based-measurement parameters (mostly geometrical: height, cross-sectional areas, and shape, microstructure homogeneity, and reflection intensity) are used for diagnostics of intraocular tissues and tumor in conventional diagnostic systems [6–8]. Statistical B-scan texture analysis-based parameters are also used for intraocular tumors [9] and thyroid tissue characterization [10].

The conventional ultrasound B-scan diagnostic systems use video (demodulated) signals to represent diagnostic images. This means that large part of information which is possibly embedded in raw or radio frequency signal (RF) representing backscattered ultrasound waves is thrown away. The information extracted from RF signals, however, could be successfully used for tissue characterization and development of quantitative ultrasound diagnostic systems [11]. For example, one-dimensional ultrasound RF signals, that is, A-scan signals are used to estimate tumor thickness, internal reflectivity, spontaneous vascular pulsation parameters [12, 13]. RF A-scan signal parameters (mean spectral frequency, the width of power spectrum, effective values of correlation function, and backscattering coefficient) can be successfully used for followup of brachytherapy treatment and characterization of malignant melanoma of choroid [14, 15].

RF ultrasound signals from B-scan-diagnostic systems were also analysed. Spectral analysis was used to obtain parameters such as the size of acoustic scatterer, acoustic concentration of scatterers, spatial variability, backscattering coefficient, attenuation coefficient and root mean square velocity fluctuation, spectral slope, and intercept. These parameters of RF signals were used as the prognostic indicators for uveal melanoma [16], correlated with microcirculatory patterns in uveal melanomas [17] and used for modeling of intraocular tumor tissues [18]. The effective scatterer size, acoustic concentration, intercept, and slope of 3D regions of interest calculated from spectral parameters of RF signals were used for characterization of cancerous lymph nodes [19] and for characterization of mammary tumors [20]. Two-dimensional spectrum analysis of RF signals was applied in ocular tumor diagnosis [21].

The literature survey shows that ultrasound RF signals are analyzed using statistical, nonparametric (Fourier), and model-based spectral analysis methods by fitting approximated backscattered spectrum model. These spectrum estimation methods suffer when data is highly nonstationary as is the case in RF ultrasound signals. Hilbert-Huang transform (HHT) [22] is a promising tool for nonstationary and nonlinear data analysis. To the best of our knowledge HHT-based methods are not yet used in the field of ultrasound-based eye tissue characterization.

The aim of this research is to develop parameterization algorithms for backscattered ultrasound RF signals received from eye tissues and to provide supplementary B-scan parametric maps, which could improve ultrasound characterization and differentiation of intraocular tissues.

## 2. RF Ultrasound Data

The hardware used for acquisition of raw RF signals comprising ultrasound B-scans was described in [23]. Briefly, the hardware system could be specified as follows. The ultrasound B-scan system is Mentor Advent A/B (Advent, Norwell, MA, USA), with mechanically scanning 15 MHz transducer. The original ultrasound scanner is supplemented with signal acquisition extension [23]. Data acquisition system was prototyped using computerized digitizer PICO 5203 (Pico Technology, Cambridgeshire, UK) having 32 MB of buffer memory, 8 bits in amplitude resolution, and 250 MHz of sampling frequency.

## 3. Algorithms for Characterization of Backscattered Signals

Empirical mode decomposition (EMD) and ensemble empirical mode decomposition (EEMD) followed by Hilbert transform (Hilbert-Huang transform) were used for synthesis of parametric maps and tissue characterization [22, 24]. Both EMD and EEMD methods extract so-called intrinsic mode functions (IMFs) from the raw RF ultrasound B-scan signals. IMFs serve as an input to Hilbert transform, which outputs analytical (complex) signals. By taking modulus and argument of complex signals. analytical amplitude and phase are extracted from each IMF. Finally, distributions of instantaneous frequency (derivative of analytical phase) and amplitude are calculated for each IMF.

In order to characterize instantaneous amplitudes and frequencies, Nakagami distribution was used since it has been found to be suitable for ultrasound signal characterization previously [25]. Nakagami distribution is parameterised by two parameters: scaling parameter Ω, which reflects distribution of signal power, and *m*, which determines the shape of the distribution. The Nakagami distribution parameters were estimated from the 1st EMD IMF and 2nd EEMD IMF. Both instantaneous amplitudes and frequencies were parameterized for all B-scan RF signal lines.

Two additional parameters, spectral slope and intercept [26], were calculated for characterization of echograms inside the regions of interest (ROI). The signals were divided into segments and then windowed using Hamming windows. Fourier transform-based estimates of power spectrum were averaged in order to reduce spectrum dispersion. The parameters (intercept and slope) were obtained after linear fitting of calibrated spectral function in frequency band 5–18 MHz. One more method to characterize nonstationary RF signal by mean instantaneous frequency (MIF) and mean instantaneous bandwidth (MIB) was used as described previously in [23].

**Figure 1:**
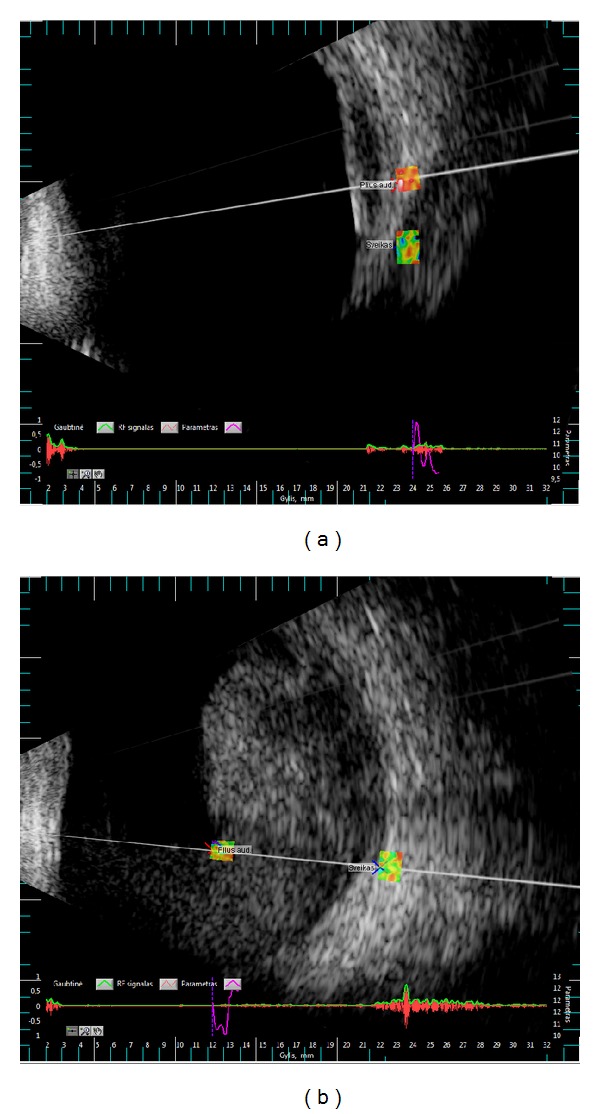
Representative examples of B-scan images with manually selected regions: (a) in healthy orbit and extraocular muscle (case no. 164), (b) in healthy orbit and intraocular blood (case no. 84).

The newly developed software allows opening and processing of raw RF ultrasound data files obtained by ultrasound diagnostic scanner. At first, RF ultrasound one-dimensional signals (A-scans) comprising B-scan sector are demodulated and mapped from sector data to raster data as a greyscale B-scan image (see grayscale images in Figure 1). Then two ROIs are selected interactively by dragging cursors. The first ROI is primarily meant to mark the suspicious tissue and the second ROI—the healthy tissue. Selected regions (matrixes of raw RF ultrasound data) are passed to parameterization algorithms. The results of parameterization by selected algorithm are added as a new layer to B-scan at locations of selected ROIs (the colored boxes in Figure 1).

RF ultrasound (B-scan) signals were registered for 57 clinical cases. An experienced ophthalmologist has selected two ROIs for each B-scan case. The size of ROIs was kept to cover the area of the image with B-scan amplitude as uniform as possible. In order to achieve uniformity of B-scan amplitude, the ROI size was varied from 1.1 mm to 1.8 mm in depth (the mean being 1.5 mm) and from 5 to 12 echoscopy lines in width (the mean being 8.6). Then the RF signals of both ROIs were processed by the parameterization algorithms, and calculated parameters were stored into the database.

## 4. Visualization of Tissue-Characterizing Parameters

The “rose” or “radar” type diagrams were used in order to present all sixteen parameters (see Table 1) in one diagram. Such presentation of parameters that characterize the tissue could be useful during visual preliminary analysis, that is, before application of automatic classification algorithms such as rule-based classifiers or neural networks.

**Figure 2:**
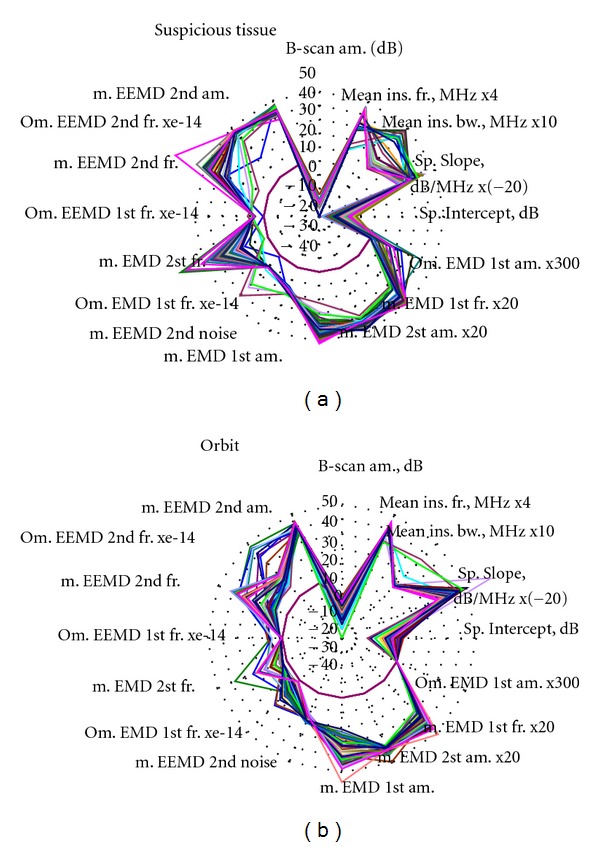
General view of all parameters characterizing: (a) suspicious tissue inside the eye, (b) healthy orbit tissue.

The whole set of 57 clinical cases of eye B-scan signals were parameterized. The general view of these parameters is presented in Figure 2. The parameter array (dimensions 57 × 16) was obtained from signals backscattered in healthy tissue of orbit, and the same size array was obtained in case of suspicious tissues inside the eyeball. 

Close analysis of diagrams in Figure 2 shows that the distributions of the parameter values are different for healthy and suspicious tissues. For example, Ω for 1st IMF and Ω for RF signal parameters are distributed widely in suspicious tissues regions, while the same parameters in healthy orbit tissues are uniformly close to zero. The wide spread of values of the parameters could be noted as common feature of signals backscattered from intraocular suspicious tissues. The smaller variability of parameters from healthy tissues of the eye orbit could be explained by uniformity and similarities of these tissues. Therefore, in future, the tissues of eye orbit could be used as the reference backscattering target of eye.

**Figure 3:**
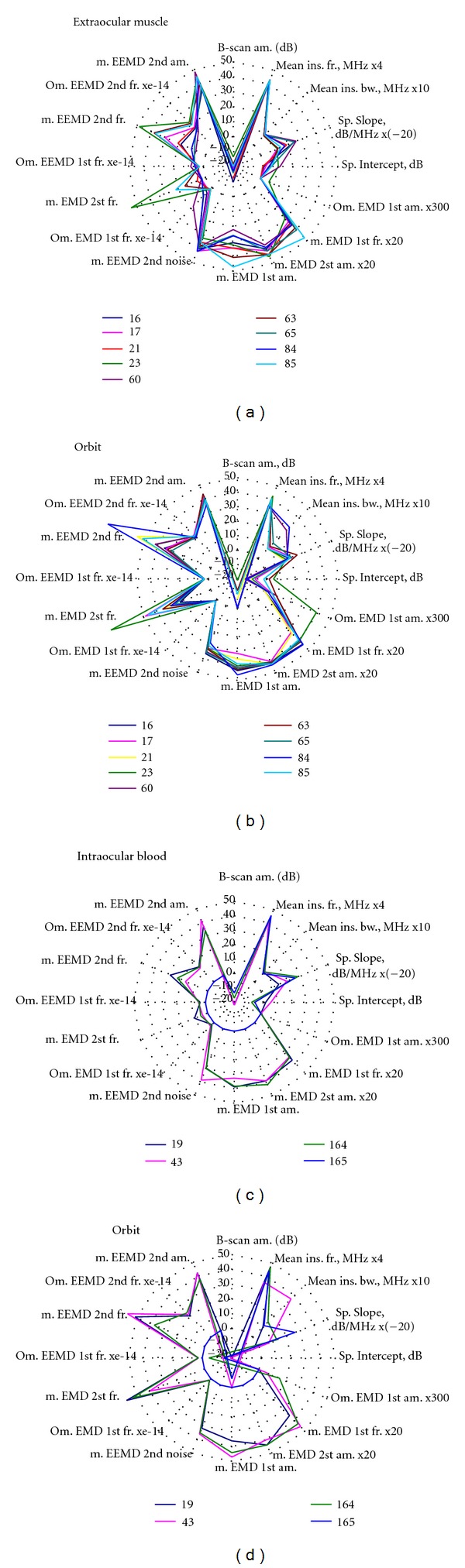
The distribution of parameter values for tissues differentiation: (a) healthy extraocular muscle (b) healthy orbit tissue (of the same eye); (c) blood inside the eyeball (d) healthy orbit tissue (of the same eye).

Several clinically confirmed cases of healthy (extraocular muscle) and pathologic (intraocular blood) tissues were analyzed in order to investigate the power of proposed technique to differentiate between types of ocular tissues. The obtained illustrative diagrams (Figures 3(a) and 3(c)) indicate some differences among parameters characterizing ultrasound signals backscattered from intraocular blood or extraocular muscle. It can be also observed that parameters estimated from healthy orbit tissues exhibit similar values and patterns of radar type diagrams (Figures 3(b) and 3(d)).

The multitude of extracted parameters makes visual analysis difficult in case of subtle differences among eye tissues. Automatic data mining analysis methods and classification techniques could potentially increase the accuracy of tissues differentiation.

## 5. Automatic Classification of Ocular Tissues

The computer software for data mining, see 5.0/C5.0 [6], was applied for automatic classification of RF ultrasound B-scan signals in the database. In total, 26 cases have been analyzed. The same sixteen parameters were used from each of 26 signals representing different clinical cases. We used predictive modeling algorithm for classification. This algorithm forms a decision tree or a set of rules understandable by a human. Classification of cases into three classes (intraocular blood, healthy orbit tissue, and extraocular muscle) was performed with decision tree of size 3, and classification error was 3.8% (1 case in dataset, see Table 2). The extraocular muscle and intraocular blood were classified without errors. The only error occurred in one case, when healthy orbit tissue was misclassified as extraocular muscle tissue. The most specific parameter for differentiation of intraocular blood was found to be spectral intercept. The Nakagami distribution *m* parameter for EMD 1st IMF of instantaneous frequencies was found to be the most specific parameter for differentiation of healthy orbit tissue from extraocular muscle. It should be mentioned that due to the small dataset, there was no possibility to test classification accuracy on new upcoming data.

## 6. Discussion

The algorithms and software for eye tissues differentiation were developed using the analysis of modulated (RF) ultrasound B-scan signals. The algorithms parameterize the RF ultrasound signals in frequency and joint time-frequency domains. The classical Fourier and relatively new Hilbert-Huang transforms were employed to characterize the signals from selected regions of eye tissues. In particular, the following parameters were calculated: B-scan envelope amplitude (dB), approximated spectral slope (dB/MHz), approximated spectral intercept (dB), mean instantaneous frequency (MHz), mean instantaneous bandwidth (MHz), and Nakagami distribution parameters *m* and Ω characterizing Hilbert-Huang transformation output. The extracted signal parameters were processed using data mining software and used to build the decision tree for automatic tissue classification. The pilot trial to automatically differentiate among *corpus vitreum* blood, extraocular muscle, and orbit tissues resulted in classification error of 3.8% in the database of 26 clinical cases of ocular tissues.

Our research is limited due to lacking of comparison with gold standard imaging modality such as MRI or with histological confirmation. However, application of the proposed method could be compared to similar research of eye tissue differentiation. In this pilot study we first evaluated differentiation of the simplest ocular tissues. As discussed by Fu et al. [4], the differentiation of eye tissues is often performed using the following ultrasonographic characteristics [4]: shape of lesion, reflectivity (low, medium, and high), internal structure consistency or irregularity, acoustic shadowing, and attenuation (from negligible up to high). However, this subjective and qualitative interpretation of B-scan images of eye tissue is hard to quantify and to use in automatic tissue differentiation algorithms. Output of our method estimates quantitatively these ultrasonographic characteristics using set of RF signals processing algorithms, similarly as was reported in [16, 19, 21]. Related study [4] proposed to use the identification of extraocular muscle as a reference to avoid misinterpretation of extrascleral growth of intraocular tumor. Internal blood was also assessed [4] as another important factor when discriminating hemorrhagic lesion from choroidal melanoma. In rare cases choroid hemangiomas may grow in spite of benign histology [27]. These pathologies were found hard to differentiate which complicates decision on the best treatment. In such cases ultrasonic followup should be provided for evaluation of changes in formation size and internal reflectivity [27]. Therefore, improvement of internal blood differentiation is important. The extremely high internal reflectivity typical for choroid hemangioma should be verified with biopsy. Fledelius [27] also has described the classical CT-scan error miss interpreting oblique section of inferior rectus muscle. Supplementary ultrasonography of external eye muscles was found valuable in ophthalmologist's evaluation. Therefore improvement of muscle differentiation is also important. Our results confirm forecasted [28] advantages of the RF-based quantitative analysis, allowing additional digital manipulation for overcoming certain limitations of qualitative interpretation. The second issue of our approach was application of complex algorithms for tissue characterization in relation with backscattering spectra model-based methods [18, 29, 30] and empirical or statistical estimation methods [25]. The backscattering models were theoretically and practically tested [29] with regard to the properties of the observed backscattering spectra. The estimated sizes of acoustic scatterers quite well correspond to the dimensions of observed histological structures. Our study showed that complex evaluation of backscattering spectra model based methods together with empirical or statistical estimation methods provides additional information and allows for better tissue characterization.

In conclusion, RF ultrasound signal analysis can be used to differentiate different ocular tissues. The critical problem in decreasing the tissues classification error is the availability of representative database having sufficient amount of annotated ultrasound data. One possible application of proposed method is the differentiation of intraocular tumors.

## Figures and Tables

**Table 1:** The list of parameterization algorithms and extracted parameters.

Title of the algorithm	Title of the parameter
Amplitude demodulation	(1) B-scan amplitude, dB

Short-time Fourier transform	(2) Mean instantaneous frequency, MHz
	(3) Mean instantaneous bandwidth, MHz

Backscattered power spectra linear approximation	(4) Spectral slope, dB/MHz
	(5) Spectral intercept, dB

Empirical mode decomposition	(6) Nakagami *m* parameter for EMD 1st IMF instantaneous frequencies
	(7) Nakagami *m* parameter for EMD 1st IMF amplitudes
	(8) Nakagami Ω parameter for EMD 1st IMF instantaneous frequencies
	(9) Nakagami Ω parameter for EMD 1st IMF amplitudes
	(10) Nakagami *M* parameter for EMD 2st IMF instantaneous frequencies
	(11) Nakagami *M* parameter for EMD 2st IMF amplitudes

Ensemble empirical mode decomposition	(12) Nakagami *m* parameter for EEMD 2nd IMF noise
	(13) Nakagami Ω parameter for EEMD 1st IMF instantaneous frequencies
	(14) Nakagami Ω parameter for EEMD 1st IMF amplitudes
	(15) Nakagami *m* parameter for EEMD 2nd IMF instantaneous frequencies
	(16) Nakagami *m* parameter for EEMD 2nd IMF amplitudes

**Table 2:** Results of automatic tissues differentiation.

	Classified as		True class
Extraocular muscle	Intraocular blood	Orbit	
9	—	—	Extraocular muscle
—	4	—	Intraocular blood
(1)	—	12	Orbit
